# Research on performance variations of classifiers with the influence of pre-processing methods for Chinese short text classification

**DOI:** 10.1371/journal.pone.0292582

**Published:** 2023-10-12

**Authors:** Dezheng Zhang, Jing Li, Yonghong Xie, Aziguli Wulamu

**Affiliations:** 1 School of Computer and Communication Engineering, University of Science and Technology Beijing, Haidian, Beijing, China; 2 Beijing Key Laboratory of Knowledge Engineering for Materials Science, University of Science and Technology Beijing, Haidian, Beijing, China; Universiti Teknikal Malaysia Melaka Fakulti Teknologi Maklumat dan Komunikasi, MALAYSIA

## Abstract

Text pre-processing is an important component of a Chinese text classification. At present, however, most of the studies on this topic focus on exploring the influence of preprocessing methods on a few text classification algorithms using English text. In this paper we experimentally compared fifteen commonly used classifiers on two Chinese datasets using three widely used Chinese preprocessing methods that include word segmentation, Chinese specific stop word removal, and Chinese specific symbol removal. We then explored the influence of the preprocessing methods on the final classifications according to various conditions such as classification evaluation, combination style, and classifier selection. Finally, we conducted a battery of various additional experiments, and found that most of the classifiers improved in performance after proper preprocessing was applied. Our general conclusion is that the systematic use of preprocessing methods can have a positive impact on the classification of Chinese short text, using classification evaluation such as macro-F1, combination of preprocessing methods such as word segmentation, Chinese specific stop word and symbol removal, and classifier selection such as machine and deep learning models. We find that the best macro-f1s for categorizing text for the two datasets are 92.13% and 91.99%, which represent improvements of 0.3% and 2%, respectively over the compared baselines.

## Introduction

Text classification is the process of determining the text category according to natural language text under the predefined category set, which means assigning predefined category tags to the text [1]. It is widely used in numerous fields, such as internet information filtering [2], question and answer topic classification [3], intelligent recommendation systems [4], sentiment analysis [5, 6], and public opinion analysis [7].

Natural language processing is one of the most important components of text classification [8]. The research context of Chinese information processing uses the computer to process and operate Chinese phonetic, shape, meaning, and other language information, including the input, output, recognition, conversion, compression, storage, retrieval, analysis, understanding [9], as well as the generation of characters, words, phrases, sentences, and chapters [10]. Currently, researchers within this field have begun to realize the importance of preprocessing during text classification tasks. In the context of multiple languages, different text preprocessing methods have emerged and have been used to study their impact on the accuracy of text classification [11]. Controlling a single text classification algorithm, researchers have studied whether different text preprocessing methods have a positive or negative effect on the classification results. However, this kind of research primarily focuses on English text with a few Chinese text classifiers being considered. Most of them only conduct experiments on a specific classification algorithm, ignoring the sensitivity of different models to different preprocessing methods. Therefore, three influential text preprocessing methods are obtained for Chinese texts through a large number of investigations: word segmentation, Chinese specific stop word removal, and Chinese specific symbol removal. The text classification algorithms that can be applied to the Chinese field are divided into three categories: simple machine learning algorithms, deep learning algorithms and algorithms based on pretraining language models. After combining the three Chinese text preprocessing methods, experiments are carried out on different text classification algorithms, in order to explore the influence of Chinese text preprocessing methods more accurately regarding the performance of text classification on the basis of model sensitivity.

The following content of the paper has been organized as follows: Section 2—The research objective for this paper. Section 3—Introduces the exploration of related work regarding preprocessing methods’ influence on the classification model. Section 4—Explains the text preprocessing methods. Section 5—Illustrates the details of the datasets and models. Section 6—Experimental results explained in detail. Section 7—Analysis of the experimental results. Section 8—Conclusions.

## Research objective

**Fig 1:**
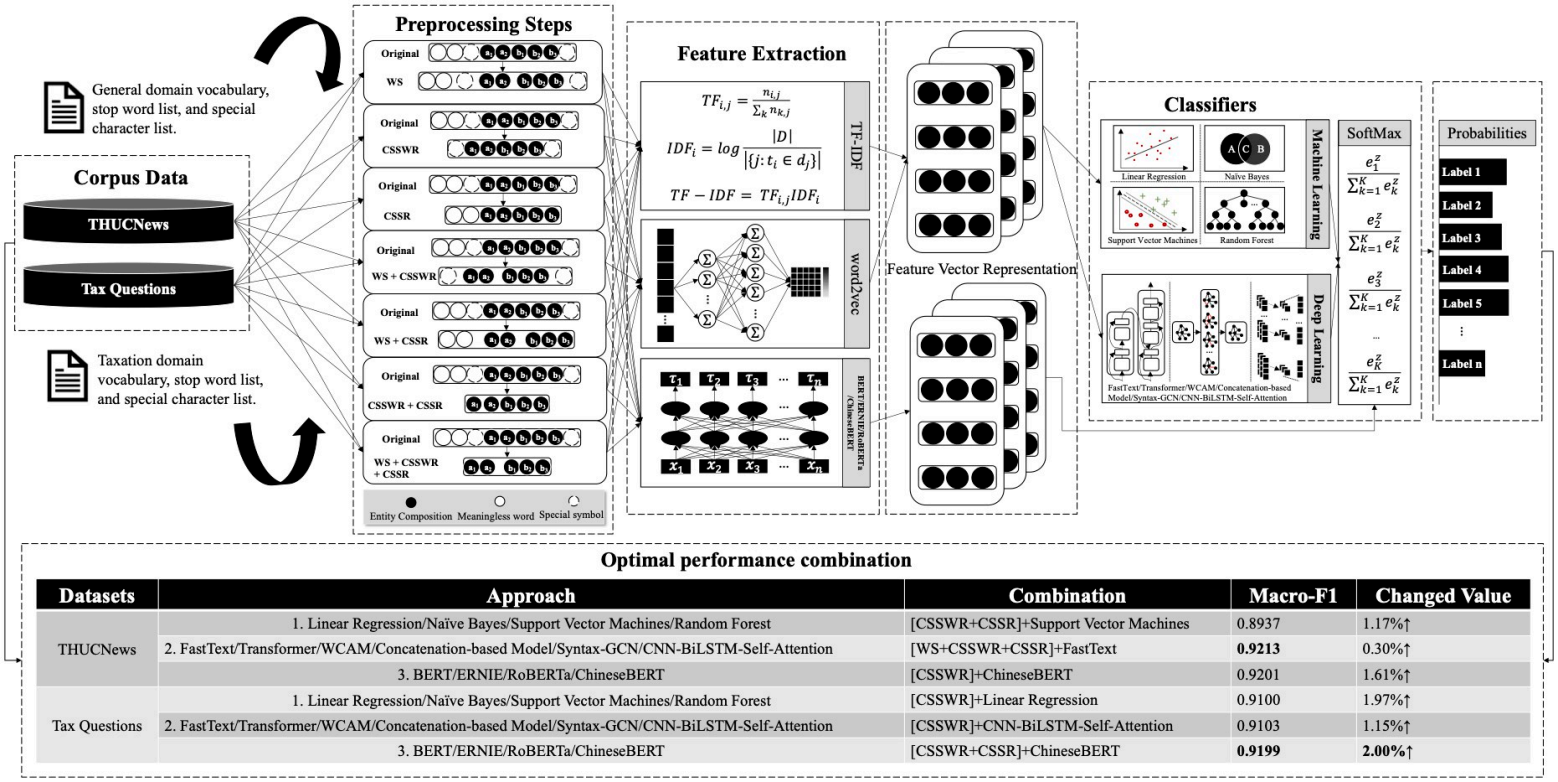
The workflow of the proposed approach.

The purpose of this study is to analyze and measure the effect of preprocessing techniques on the performance of Chinese text classification models. The overall workflow of this paper is shown in Fig 1.

We explore the influence of the proposed widely utilized preprocessing methods for Chinese text that include word segmentation (WS), Chinese specific stop word removal (CSSWR), and Chinese specific symbol removal (CSSR), in a divergent mode. All possible combinations of the preprocessing methods of the fifteen different classifiers are considered, which are machine learning classifiers, deep learning classifiers, and pretrained language model-based classifiers. Therefore, the effort on the part of the referring preprocessing methods in regard to the success of Chinese classification concerning the potential influencers among these methods as well as the methods of habituation to the models are discussed. In order to elucidate the variations of this work from previous ones, the analytic preprocessing methods and experimental conditions are illustrated in Table 1. The experimental conditions are the preprocessing methods, the language, the considered classifiers, and the results.

**Table 1:** Comparison of the considered condition with previous research.

Paper	Preprocessing Methods	Language	Considered Classifiers	Result
[12]	Rare word removal, Blacklist word unification, Stop word removal, stemming	English	Simple machine learning and deep learning classifiers	Different combinations improve the F1 between 0.0211 and 0.0248
[13]	Noise data filter, Incomplete data processing, Standardization, Symptom specification	Chinese	-	-
[14]	Stop word removal, Porter stemmer, Lemmatization	Spanish, English	Simple machine learning classifiers	Applying methods such as stop words to long datasets has positive effect.
[15]	Lowercase, Punctuation removal, Number removal, Slang and abbreviation removal	English	Simple machine learning and deep learning classifiers	The accuracy improves between 0.0130 and 0.0146.
[16]	Tokenization, Stop word removal, Repeated letter removal, Tashkeel removal	Arabic	Simple machine learning classifiers	Stop words decreases the performance; Stemming increase the results.
[17]	Word Segmentation, Useless symbols andstop word removal	Chinese	Deep learning classifiers	-
[18]	Word Segmentation, Stop word removal, Word frequency statistics	Chinese	Simple machine learning classifiers	Preprocessing method helps the classification performance.
[19]	Spelling correction, Lowercase, Html tags, Repeated characters	English	Simple machine learning classifiers	Classification accuracy improves around 0.0148.
[20]	Invalid data removal, Deduplication removal, Symbol cleaning	Chinese	-	Propose the effective preprocessing strategy.
[21]	Redundant removal, Dose normalization, Standarzation of units	Chinese	-	Data after preprocessing can be effectively mined.
[22]	Tokenization, Stop word removal, Lemmatization, Stemming	English	Simple machine and deep learning classifiers	-

The contributions of this paper are as follows:

- We make an effort to explore the combinations from the aspects of the preprocessing methods like word segmentation, Chinese specific stop word removal and Chinese specific symbol removal, feature extraction methods like TF-IDF, word2vec, pre-trained language model and classifier selection like machine learning model, deep learning model based on general and tax areas.
- The experiments are considered comparatively on two datasets including THUCNews and tax questions.
- Find the best combination of preprocessing and classification models for categorizing text for THUCNews and Tax questions: FastText with WS, CSSWR and CSSR, ChineseBert with CSSWR and CSSR, respectively. And the macro-f1s of them are 92.13% and 91.99% which improved 0.3% and 2%, respectively.

## Related work

For the Chinese text classification task, researchers have explored many algorithms designed to resolve semantic understanding problems such as text representation, feature selection, and extraction [23]. From these Chinese text classification achievements, we conclude three common types of models machine learning, deep learning, and networks based on pretraining language models.

Common simple machine learning models used for Chinese text classification are SVM [24–26], K-nearest Neighbor [27], Random Forest [28], and Naïve Bayes [29]. To adapt to Chinese texts, many improved simple machine learning models have emerged to improve the classification effect of Chinese texts. Novel loss functions and the introduction of new features can add to the effects of such models [30, 31]. Although such classification models are simple and effective, their accuracies are not high, and they are not the first choice if applied to actual industrial applications.

Deep learning models have been populated for years and many researchers have tried to explore techniques to resolve the Chinese classification problem with improved results [32]. As a result, the Chinese text feature becomes the research key point in order to improve the performance. Character, word, and sentence level features clearly represent the semantic information of Chinese text. For example, RAFG [33] applies a serialized BLSTM structure to model the sequence characteristics of the Chinese text. The similar structure of BiGRU has also been considered, and it combines Chinese grammar rules in the form of constraints and simulates the linguistic functions of the target sentence by standardizing the output of adjacent positions [34]. Additionally, the hybrid attention network applies CNN and RNN to extract the semantic features of Chinese text by capturing class-related attentive representation from the word and character level features simultaneously [35]. Furthermore, the fusion of these features can also improve performance [36]. The researchers explore multi-strategies to implement a better integration. For example, the LSTM, BiLSTM, CNN, and TextCNN features are popular for extracting Chinese semantic [37, 38]. WCAM [39] intelligently utilizes the attention model to integrate character-level and word-level features used to represent the semantic relationship between the Chinese text. Graph convolution networks also enhances the understanding of the diverse grammatical features of Chinese microblogs for emotion classification [40]. Although, the Chinese text classification model based on deep learning is better than being based on simple machine learning, it still has the limitation that the classification effect changes with the complexity of the model. In order to allow the model to fully study and comprehend the text semantic characteristics, to help improve the effect of classification, some preliminary training language models have emerged.

Pretraining language models have frequently been applied in large-scale corpus scenarios. For example, researchers use BERT and domain specific corpora for traditional Chinese medicine clinical record classification and have shown great results [41]. Extensive experiments on a hybrid model that combines BiGRU and BERT have demonstrated that the Chinese sentiment classification can generate new insights for future development [34]. ERNIE is a pre-trained language model for Chinese corpus. Question classification has been resolved by considering the ERNIE pretraining model together with feature fusion [42]. Specifically, RoBERTa [43] has been implemented and fine-tuned for Chinese text classification, and ChineseBERT [44] incorporates both Chinese character glyph and pinyin information. Although the pre-trained language models can achieve good results during most text tasks, the training cost of these methods is extremely high, requiring a large amount of text corpus, compared to the simple and the deep learning models.

Among all the methods used to improve the Chinese classification effect, text preprocessing is an essential step. In addition to optimizing the model structure, proper Chinese pretreatment methods can effectively improve the performance. Many researchers have focused on exploring the effects of text preprocessing on text classification. However, scholars have currently been studying the preprocessing methods influence on English text classification accuracy. For example, many English preprocessing methods have been explored such as removing the rare word [12], using regular expressions for blacklisted words [12], spelling correction [19], HTML tag removal [15, 19], tokenization [45], PoS tagging [12, 45], stemming [15, 45], and removing Unicode strings and noise [15]. However, they primarily studied the influence of machine and deep learning models like SMO (a variant of SVM) [19], and Multilayer Perceptron [45]. The text preprocessing influence on Arabic emotion analysis task results has been introduced [16]. At the same time, the influence of text preprocessing methods on Spanish and English text classification has also attracted significant research attention [14].

The existing literature explored more on English data and other language data. And most of them focus on the number of preprocessing methods and use a single type of feature extraction and classification models to conduct experiments and draw conclusions, ignoring the relationship between different feature extraction methods and classification models and preprocessing methods [13, 17, 18, 20, 21]. Some common Chinese preprocessing methods include Word segmentation [17, 18], Useless symbols and stop word removal [13, 17, 18, 20], Redundancy removal [21], Dose normalization [17, 18], Standardization of units [17, 18], Incomplete data processing [13], Standardization [13], Deduplication removal [20], and Word frequency statistics [18]. In order to solve the problem of exploring the influence relationship of preprocessing methods, feature extraction methods, and classification models in different area, we choose THUCNews [46] and tax question as our research datasets. Therefore, we obtain the optimal combination of preprocessing methods and feature extraction and classification models for the two datasets. And it will provide a reference value for the selection of preprocessing methods for other Chinese text categorization tasks in the future.

## Preprocessing methods

The Chinese text preprocessing methods used in this paper are word segmentation (WS), Chinese specific stop word removal (CSSWR), and Chinese specific symbol removal (CSSR). Chinese word segmentation is a necessary step to process Chinese text into discrete word representations that can be understood by the model. Without segmentation, the model struggles to directly comprehend continuous Chinese characters directly. Word segmentation can better express sentence structures and semantic information, enabling the model to better understand Chinese text better and improve classification accuracy. In addition, Chinese stopwords do not contribute to text classification tasks and can increase computational costs during model training and prediction. Removing stopwords can thus reduce the dimensionality of the feature space, contributing to improved model generalization ability and efficiency. Furthermore, Chinese Special characters usually do not carry semantic information, and removing them can purify the text data, enhancing classification accuracy and stability. Finally, data cleaning ensures input data quality, improving the model’s robustness and generalization ability. Therefore, we choose these three typical preprocessing methods to explore their influence on Chinese text classification performance.

For Chinese text preprocessing, WS is an indispensable step. It is the process of regrouping successive word sequences according to certain norms. We know that the natural delimiter between words can be represented as a space in English writing. The Chinese word, sentence, and paragraph can simply be represented by obvious delimiter demarcations; however, it cannot be represented in this way due to the form of Chinese words. There are abundant deformations in English words. To cope with these complex transformations, English NLP has some unique processing steps compared to Chinese, which are called Lemmatization and Stemming extraction. Their exists problems in the division of English phrases. Chinese is much more complex and more difficult than English regarding word layer. For example, Chinese word segmentation needs to consider the granularity problem, the larger the granularity, the more accurate the meaning of the expression, leading to less recall. Therefore, Chinese requires different scenarios and requires different granularity. Common Chinese word segmentation tools primarily include Hanlp, jieba, and Stanford tokenization, while Gensim, NLTK, and Keras are often used as English word segmentation. In this paper, Jieba is primarily used as an auxiliary tool for Chinese word segmentation.

CSSWR contains not only the common Chinese stop words like pronouns, prepositions, conjunctions, interjections, and onomatopoeias, but also the meaningless Chinese phrases. According to our dataset, the specific phrases like “please (请问)” and “hello (你好)” are identified as situational language.

CSSR refers to the varied punctuation in the Chinese grammar database. For example, “《》” represents a book or article in Chinese. It has not been defined in English since the title of the book or newspaper has been represented in italic or underlined. “、” represents the meaning of a stop sign and plays the role of dividing the parallel elements into sentences. There is no stop sign in English, and commas are often used for parallel elements in segmented sentences. Chinese has a space sign “·”, which is used in the middle of words that need to be separated, such as month and date, transliterated first and last name.

In this paper, all combinations of Chinese text preprocessing methods have been considered in Table 2. Word segmentation is represented as WS, Chinese specific stop word removal is represented as CSSWR and Chinese specific symbol removal is represented as CSSR. All of the text preprocessing methods have two statuses which are true (T) and false (F). T means application of the method and F represents text processing without the specific method.

**Table 2:** Combinations of Chinese preprocessing methods.

Number	Preprocessing Method	With WS (T) | Without WS (F)	With CSSWR (T) | Without CSSWR (F)	With CSSR (T) | Without CSSR (F)
0	None	F	F	F
1	Pre_1	T	F	F
2	Pre_2	F	T	F
3	Pre_3	F	F	T
4	Pre_4	T	T	F
5	Pre_5	T	F	T
6	Pre_6	F	T	T
7	Pre_7	T	T	T

## Experimental setup

### Dataset description

The first dataset used in this paper is collected from our project. Our dataset primarily contains user questions about the tax field and the corresponding scenario categories. We abstracted the category labels to numbers for model training. Most of these questions are in the form of interrogative sentences, for example “Hello, could I ask about how to fill in the export tax refund filling form? (你好, 想问一下出口退免税备案表怎么填写?)”. The scenario category is determined by the data provider. Furthermore, one of the biggest features of this dataset is it contains a large number of daily expressions, for example “Hello (你好)”, “Could I ask?”, “Could I ask? (咨询一下)”, and “Could I ask? (方便问一下)”. However, such daily words are meaningless in Chinese. Therefore, they are defined as stop words in this study.

In addition, another feature of this dataset is the inclusion of some technical terms, for example “tax refund (退免税)” in “Hello, could I ask about how to fill in the export tax refund filling form? (你好, 想问一下出口退免税备案表怎么填写?)”, or “Differential taxation (差额征税)” in “How to calculate the difference tax for taxpayer who provide travel services? (纳税人提供旅游服务选择差额征税怎么计算?)”. Such professional words may cause the phenomenon of wrong sentence breaking in Chinese if there is no relevant background knowledge, thus, affecting the sentence understanding accuracy. The last feature is the special punctuation in this dataset, for example “《》” in “How to fill in the contact person on the” Report on Cross-Region Tax-related Matters”? (《跨区域涉税事项报告表》上联系人如何填写?)”. In Chinese, “《》” is often used to refer to specific names such as books or articles, and in this dataset, it is used to refer to tax-related file names or legal provisions. This type of notation does not exist in other languages such as English, which uses double quotes to refer to specific names.

In our dataset, there are a total of 34474 samples including 56 classes, the details are shown in Tables 3 and 4. A subset of the dataset is used to train the network (27579 samples: 80% training dataset), and the remaining data (6895 samples: 20%) is used to test and validate the network. There are 3446 specific samples in the test dataset and 3449 samples in the validation dataset. In the training, test, and validation datasets, there are two parts: sentence and label.

**Table 3:** Tax question dataset.

Label	Class Name	Training	Test	Validation	Total
0	Individual Income Tax	150	19	31	200
1	Report Tax	752	86	100	938
2	Differential Taxation	242	29	29	300
3	House Tax	437	59	63	559
4	Medical Treatment	126	14	11	151
5	Address Changing	51	10	5	66
6	Receipt	1076	134	144	1354
7	Real Estate Sales	590	62	73	725
8	Real Estate Gift	921	114	112	1147
9	Couple’s Property Changing	187	21	27	235
10	Personal Property Inheritance	490	85	63	638
11	Personal Income Tax	24	7	1	32
12	Personal Income Tax Fee Refund	500	59	47	606
13	Personal Income Tax APP Operation	379	40	51	470
14	Personal Income Tax APP Download	776	97	90	963
15	Salary	246	29	30	305
16	Stock Transfer	51	7	3	61
17	Equity Transfer	498	67	81	646
18	Depreciation of Fixed Assets	168	21	22	211
19	Advertising Expenses and Business Publicity Expenses	533	60	64	657
20	Domestic Passenger Transport deduction	339	47	51	437F
21	Verification Collection	503	47	85	635
22	Continuing Education	217	28	34	279
23	Slash Taxes and Fees	48	6	3	57
24	Simple Construction Tax	373	34	53	460
25	Bonus Individual Income Tax Calculation	700	98	86	884
26	Energy Conservation and Environmental Protection	714	78	71	863
27	Donation Scenario	375	53	45	473
28	Number of Years for Making Up Losses Carried Forward	945	122	113	1180
29	Tax Credit Rating	362	54	43	459
30	Issue of Tax Payment Certificate	172	22	15	209
31	Agriculture, Forestry, Animal Husbandry and Fishing Concessions	123	17	16	156

**Table 4:** Tax question dataset.

Label	Class Name	Training	Test	Validation	Total
32	Other	6685	819	833	8337
33	Change of Enterprise Information	48	4	6	58
34	Enterprise Cancellation	151	18	19	188
35	Preferential Treatment for Underdeveloped Areas and Old Revolutionary Base Areas	220	23	30	273
36	Financing Sale and Leaseback	153	11	22	186
37	Finance Lease	179	18	24	221
38	Preferential Treatment for Software Enterprises	1235	174	151	1560
39	Elderly Support	290	35	33	358
40	Preferential Treatment for Ethnic Minority Areas	141	27	11	179
41	Social Insurance Premium Waiver	537	65	68	670
42	Tax Control Equipment	138	13	20	171
43	Tax Registration	459	64	63	586
44	Sales of Used Fixed Assets	259	32	38	329
45	Tax Incentives for Small and Micro Businesses	246	24	30	300
46	Research and Development Expenses Deduction	238	27	27	292
47	General Taxpayer to Small-Scale Taxpayer	432	48	48	528
48	Epidemic Prevention and Control	729	98	95	922
49	Comprehensive Income	64	9	9	82
50	Time of Occurrence of VAT Tax Liability	147	19	22	188
51	Hire the Preferential	935	105	92	1132
52	Home Loan Interest	255	40	26	321
53	Housing Rents	163	30	19	212
54	Children’s Education	478	63	63	604
55	Special Additional Deduction Items	329	54	38	421
Total		27579	3446	3449	34474

The second dataset we used is THUCNews [46] which is a public dataset. This dataset is based on the generation of historical data filtering of the Sina news RSS subscription channel from 2005 to 2011 and contains 200,000 news documents, all in UTF-8 plain text format, the details are shown in Table 5. On the basis of the original Sina news classification system, the Natural Language Processing Laboratory of Tsinghua University reintegrated and divided 10 candidate categories: finance, realty, stocks, education, science, society, politics, sports, games, and entertainment.

**Table 5:** THUCNews dataset.

Label	Class Name	Training	Test	Validation	Total
0	finance	18000	1000	1000	20000
1	realty	18000	1000	1000	20000
2	stocks	18000	1000	1000	20000
3	education	18000	1000	1000	20000
4	science	18000	1000	1000	20000
5	society	18000	1000	1000	20000
6	politics	18000	1000	1000	20000
7	sports	18000	1000	1000	20000
8	game	18000	1000	1000	20000
9	entertainment	18000	1000	1000	20000
Total		180000	10000	10000	200000

### Feature extraction

In our study, the extraction of each text feature is different for different classifiers. For simple machine learning classifiers, the Term Frequency—Inverse Document Frequency (TF-IDF) method is chosen. TF-IDF [47], characterized as term recurrence reverse record recurrence, is utilized to figure out what expressions of a corpus might be ideal to utilize for quantifying the significance of words in a particular document or piece of text. Since there exists different ways of deciding term recurrence, we refer to the crude recurrence of a term in a document, given as follows:

$$\begin{array}{c} {\text{TF}}_{i,j}=\frac{n_{i,j}}{\sum_{k}\;n_{k,j}}, \end{array}$$ (1)

$$\begin{array}{c} {\text{IDF}}_{i}=log\frac{\left|D\right|}{|j:t_{i}\in d_{j}|}, \end{array}$$ (2)

$$\begin{array}{c} \text{TF}-\text{IDF}={\text{TF}}_{i,j}{\text{IDF}}_{i}, \end{array}$$ (3)

where *n*_i,j_ is the number of occurrences of the word in the document, ∑_*k*_ n_k,j_ is the sum of the occurrences of all words in the document, |*D*| is the total number of files in the corpus and |*j*: t_i_ ∈ d_j_| is the number of files containing the particular word.

For deep learning classifiers, the technique used to train the word vectors, word2vec, carries out two models that take tokenized text and determine a component vector for each sort in this informational index. For this paper we utilized the consistent skip-gram model, a neural network model that maintains a strategic distance from various secret layers to permit incredibly quick and productive preparation as compared to most grouping calculations. During preparation, each word in the informational collection is utilized as a contribution to a log-direct classifier, which learns word portrayals by attempting to foresee words happening inside a specific reach to one or the other side of the word. The skip-gram model is used by default and has the training complexity architecture of

$$\begin{array}{c} Q=M\times (N+N\times {\text{log}}_{2}\left(Y\right)) \end{array}$$ (4)

where the maximum distance for words is M, N is word representations, and Y is dimensionality. For pre-training representation model, a fine-tuning step is needed to tokenizer the function, especially when the input text is preprocessed.

### Experimental configuration

The operating system used in this experiment was Ubuntu 20.04.2 LTS, the programming language was python3.7, the deep learning framework was Pytorch, the graphics card was one Nvidia GeForce RTX 3070 with 8GB of memory, and the CUDA version was 11.0. The optimizer selected was Adam. Given a dataset as an input, Python’s NLTK was used and a new file was created as output for each pre-processing technique. For the preprocessing methods of the WS, CSSWR, and CSSR in the general domain, we primarily utilized the jieba tool. Additionally, we have incorporated manually summarized components, including specialized vocabulary lists, stop word lists, and special character lists tailored to the field of taxation.

### Classifiers

Among the many available text classifiers, we investigated fifteen popular machine learning, deep learning, and pre-trained language models within the last five years as follows:

CNN-BiLSTM-Self-Attention [37]. Integrates two semantic features CNN, and BiLSTM based on self-attention mechanisms.

Concatenation-based Model [38]. Integrates three semantic features TextCNN, BiLSTM, and LSTM for Chinese text.

WCAM [39]. Integrates two attention model levels: word-level attention model captures salient words, and character-level attention model selects discriminative Chinese text characters.

Syntax-GCN [40]. A syntax-based graph convolution network model enhances the diverse grammatical structure understanding of Chinese microblogs.

RoBERTa [43]. Is adopted and fine-tuned for Chinese text classification. The model is able to classify Chinese texts into two categories, containing descriptions of legal behavior and descriptions of illegal behaviors.

ChineseBERT [44]. Incorporates both the glyph and pinyin information of Chinese characters into a language pretraining language model. Linear Regression (LR). Is a popular algorithm that belongs to the Generalized Linear Model methods and is also known as Maximum Entropy [48].

Naive Bayes (NB). Is a simple but powerful linear classifier and is often applied with the TF-IDF feature. It is the grade factor feature for Chinese information classification [49].

Support Vector Machines (SVM). SVM is often considered as the novel proposed model with optimization in resolving text classification questions [10].

Random Forest (RF). RF operates by constructing a multitude of decision trees during training time and output classification for the case at hand [32].

DPCNN [50]. Is a word level-based network that can extract long-distance text dependencies by deepening the network.

FastText [51]. Includes using word and N-gram bags to represent statements, as well as using subword and sharing information between categories through hidden representations.

Transformer [52]. Transformer consists of self-attention and feed forward neural networks. A trainable neural network based on a transformer can be built in the form of stacked transformers.

BERT [53]. A pretrained language model that has a strong language representation ability and feature extraction.

ERNIE [54]. Is a pretrained language model based on BERT for Chinese corpus learning and consists of three-level masks: word, phrase, and entity level masking.

## Experiments and results

The performance of the experiments has been evaluated based on the following parameters.

$$\begin{array}{c} Precision=\frac{TP}{TP+FP}, \end{array}$$ (5)

$$\begin{array}{c} Recall=\frac{TP}{TP+FN}, \end{array}$$ (6)

$$\begin{array}{c} F1=2*\frac{precision*recall}{precision+recall}, \end{array}$$ (7)

$$\begin{array}{c} F1_{\text{Macro}}=\frac{\sum_{1}^{n}F1_{{\text{class}}_{n}}}{n}, \end{array}$$ (8)

where *TP* represents the sentences, which are labeled as positive and are also predicted as positive, *TN* means the sentences that are originally labeled as positive but are predicted as negative, *FP* represents the sentences, which are labeled as negative but predicted as positive, *FN* refers to the sentences which are labeled as negative and predicted as negative, and *N* and *n* mean the class number in different cases.

During the experiments, we explored all possible combinations of the three preprocessing methods. Approximately fifteen classifiers are investigated in this work.

Table 6 shows the classification performance of the four simple machine learning models in combination with different preprocessing methods on the tax question and THUCNews datasets. The results indicate that the model’s classification performance improved after combining it with the corresponding preprocessing methods, and the bolded results represent the best results for the two datasets. The best preprocessing method and model combination for the tax question dataset was linear regression with CSSWR, which attained a macro-f1of 91.00%, 1.97% better than without the preprocessing method, and the best preprocessing method and model combination for the THUCNews dataset was SVM with CSSWR and CSSR, which attained a macro-f1of 89.37%, 1.17% higher than without the preprocessing method.

**Table 6:** Macro-F1 results comparison of four widely used machine learning models under seven combinations of preprocessing methods (TQ-tax question dataset; TC-THUCNews).

	TQ	Change	TC	Change
LR	0.8903	-	0.8800	-
LR+Pre_1	0.8957	0.54%↑	0.8801	0.01%↑
**LR+Pre_2**	**0.9100**	**1.97%**↑	0.8899	0.99%↑
LR+Pre_3	0.9027	1.24%↑	0.8803	0.03%↑
LR+Pre_4	0.8990	0.87%↑	0.8771	0.29%↓
LR+Pre_5	0.8898	0.05%↓	0.8779	0.21%↓
LR+Pre_6	0.8899	0.04%↓	0.8692	1.08%↓
LR+Pre_7	0.9090	1.87%↑	0.8878	0.78%↑
RM	0.8967	-	0.8883	-
RM+Pre_1	0.8925	0.42%↓	0.8801	0.82%↓
RM+Pre_2	0.8998	0.31%↑	0.8900	0.17%↑
RM+Pre_3	0.9023	0.56%↑	0.8899	0.16%↑
RM+Pre_4	0.9057	0.90%↑	0.8920	0.37%↑
RM+Pre_5	0.9017	0.50%↑	0.8912	0.29%↑
RM+Pre_6	0.9019	0.52%↑	0.8891	0.08%↑
RM+Pre_7	0.8930	0.37%↓	0.8880	0.03%↓
SVM	0.8930	-	0.8820	-
SVM+Pre_1	0.8728	2.02%↓	0.8831	0.11%↑
SVM+Pre_2	0.8776	1.54%↓	0.8790	0.30%↓
SVM+Pre_3	0.8802	1.28%↓	0.8622	1.98%↓
SVM+Pre_4	0.8878	0.52%↓	0.8712	1.08%↓
SVM+Pre_5	0.8889	0.41%↓	0.8910	0.90%↑
**SVM+Pre_6**	0.8932	0.02%↑	**0.8937**	**1.17%** ↑
SVM+Pre_7	0.8931	0.01%↑	0.8832	0.12%↑
NB	0.8742	-	0.8822	-
NB+Pre_1	0.8870	1.28%↑	0.8931	1.09%↑
NB+Pre_2	0.8860	1.18%↑	0.8820	0.02%↓
NB+Pre_3	0.8856	1.14%↑	0.8822	-
NB+Pre_4	0.8795	0.53%↑	0.8812	0.10%↓
NB+Pre_5	0.8774	0.32%↑	0.8910	0.88%↑
NB+Pre_6	0.8811	0.69%↑	0.8837	0.15%↑
NB+Pre_7	0.8843	1.01%↑	0.8924	1.02%↑

Similarly, Tables 7 and 8 present the classification performance of the seven deep learning models after combining different preprocessing methods on the tax question and THUCNews datasets. Once again the results indicate that the model’s classification performance improved after combining it with the corresponding preprocessing methods, and the bolded results represent the best results for the two datasets. The best preprocessing method and deep learning model combination for the tax question dataset was CNN-BiLSTM Self Attention with CSSWR, which attained a macro-f1of 91.03%, 1.15% better than without the preprocessing method, and the best preprocessing method and deep learning model combination for the THUCNews dataset was FastText with WS, CSSWR, and CSSR, which attained a macro-f1of 92.13%, 0.3% improvement over not using the preprocessing.

**Table 7:** Macro-F1 results comparison of seven widely used deep learning models under seven combinations of preprocessing methods (TQ-tax question dataset; TC-THUCNews).

	TQ	Change	TC	Change
DPCNN	0.8699	-	-	-
DPCNN+Pre_1	0.8607	0.92%↓	-	-
DPCNN+Pre_2	0.8770	0.71%↑	-	-
DPCNN+Pre_3	0.9027	**3.28%** ↑	-	-
DPCNN+Pre_4	0.8504	1.95%↓	-	-
DPCNN+Pre_5	0.8538	1.61%↓	-	-
DPCNN+Pre_6	0.8788	0.89%↑	-	-
DPCNN+Pre_7	0.8781	0.82%↑	-	-
FastText	0.8876	-	0.9183	-
FastText+Pre_1	0.8926	0.50%↑	0.8877	3.06%↓
FastText+Pre_2	0.8965	0.89%↑	0.9191	0.08%↑
FastText+Pre_3	0.8888	0.12%↑	0.9187	0.04%↑
FastText+Pre_4	0.8816	0.60%↓	0.8997	1.86%↓
FastText+Pre_5	0.8897	0.21%↑	0.9188	0.05%↑
FastText+Pre_6	0.8953	0.77%↑	0.8943	0.24%↓
**FastText+Pre_7**	0.8988	1.12%↑	**0.9213**	0.3%↑
Transformer	0.8327r	-	0.8115	-
Transformer+Pre_1	0.8441	1.14%↑	0.7937	1.78%↓
Transformer+Pre_2	0.8559	2.32%↑	0.8162	0.47%↑
Transformer+Pre_3	0.8357	0.30%↑	0.7864	2.51%↓
Transformer+Pre_4	0.8420	0.93%↑	0.7424	6.91%↓
Transformer+Pre_5	0.8306	0.21%↓	0.7409	7.06%↓
Transformer+Pre_6	0.8317	0.10%↓	0.7700	4.15%↓
Transformer+Pre_7	0.8430	1.03%↑	0.7424	6.91%↓
WCAM	0.8680	-	0.9154	-
WCAM+Pre_1	0.8699	0.19%↑	0.9166	0.12%↑
WCAM+Pre_2	0.8797	1.17%↑	0.9176	0.22%↑
WCAM+Pre_3	0.8692	0.12%↑	0.9150	0.04%↓
WCAM+Pre_4	0.8728	0.48%↑	0.9081	0.73%↓
WCAM+Pre_5	0.8628	0.52%↓	0.9163	0.09%↑
WCAM+Pre_6	0.8712	0.32%↑	0.8998	1.56%↓
WCAM+Pre_7	0.8679	0.01%↓	0.9081	0.73%↓
Concatenation-based Model	0.8831	-	0.9000	-
Concatenation-based Model+Pre_1	0.8790	0.41%↓	0.8998	0.02%↓

**Table 8:** Macro-F1 results comparison of seven widely used deep learning models under seven combinations of preprocessing methods (TQ-tax question dataset; TC-THUCNews).

	TQ	Change	TC	Change
Concatenation-based Model+Pre_2	0.8904	0.73%↑	0.9103	**1.03%** ↑
Concatenation-based Model+Pre_3	0.8812	0.19%↓	0.9012	0.12%↑
Concatenation-based Model+Pre_4	0.8799	0.32%↓	0.8952	0.48%↓
Concatenation-based Model+Pre_5	0.8879	0.48%↑	0.8979	0.21%↓
Concatenation-based Model+Pre_6	0.8810	0.21%↓	0.8922	0.78%↓
Concatenation-based Model+Pre_7	0.8829	0.02%↓	0.8972	0.28%↓
Syntax-GCN	0.8997	-	0.9038	-
Syntax-GCN+Pre_1	0.8889	1.08%↓	0.8926	1.12%↓
Syntax-GCN+Pre_2	0.8999	0.02%↑	0.9073	0.35%↑
Syntax-GCN+Pre_3	0.8975	0.22%↓	0.8933	1.05%↓
Syntax-GCN+Pre_4	0.8987	0.10%↓	0.8949	0.89%↓
Syntax-GCN+Pre_5	0.8998	0.01%↑	0.9019	0.19%↓
Syntax-GCN+Pre_6	0.8933	0.64%↓	0.8812	2.26%↓
Syntax-GCN+Pre_7	0.8797	2.00%↓	0.8949	0.89%↓
CNN-BiLSTM-Self-Attention	0.8988	-	0.9096	-
CNN-BiLSTM-Self-Attention+Pre_1	0.8980	0.08%↓	0.9076	0.20%↓
**CNN-BiLSTM-Self-Attention+Pre_2**	**0.9103**	1.15%↑	0.9113	0.17%↑
CNN-BiLSTM-Self-Attention+Pre_3	0.9008	0.20%↑	0.9096	-
CNN-BiLSTM-Self-Attention+Pre_4	0.8965	0.23%↓	0.9084	0.12%↓
CNN-BiLSTM-Self-Attention+Pre_5	0.9054	0.66%↑	0.9107	0.11%↑
CNN-BiLSTM-Self-Attention+Pre_6	0.9041	0.53%↑	0.9112	0.16%↑
CNN-BiLSTM-Self-Attention+Pre_7	0.8962	0.26%↓	0.9077	0.19%↓

Next, Table 9 shows the classification performance of the four pre-trained models in combination with different preprocessing methods on the tax question and THUCNews datasets, where the results indicate that the model’s classification performance improved after combining it with the corresponding preprocessing methods, and the bolded results represent the best results for the two datasets. For the tax question dataset, the best preprocessing method and pre-training learning model combination was ChineseBert with CSSWR and CSSR, which attained a macro-f1of 91.99%, 2% increase over the case without the preprocessing method, and the best combination for the THUCNews dataset was also ChineseBert but with CSSWR, which attained a macro-f1of 92.01%, 1.61% higher than without the preprocessing method. From the above results, we conclude that the best preprocessing methods and model combinations for the two datasets were FastText with WS, CSSWR, and CSSR, and ChineseBert with CSSWR and CSSR, for the THUCNews and tax question datasets, respectively.

**Table 9:** Macro-F1 results comparison of four widely used pre-training learning models under seven combinations of preprocessing methods (TQ-tax question dataset; TC-THUCNews).

	TQ	Change	TC	Change
BERT	0.8709	-	0.8940	-
BERT+Pre_1	0.8744	0.35%↑	0.8944	0.04%↑
BERT+Pre_2	0.8473	2.36%↓	0.8836	1.04%↓
BERT+Pre_3	0.8676	0.33%↓	0.8868	0.72%↓
BERT+Pre_4	0.8682	0.27%↓	0.8208	6.96%↓
BERT+Pre_5	0.8772	0.63%↑	0.8993	0.53%↑
BERT+Pre_6	0.8805	0.96%↑	0.9025	0.85%↑
BERT+Pre_7	0.8737	0.28%↑	0.8808	1.32%↓
ERNIE	0.8877	-	0.9001	-
ERNIE+Pre_1	0.8967	0.90%↑	0.8991	0.10%↓
ERNIE+Pre_2	0.8985	1.08%↑	0.9017	0.16%↑
ERNIE+Pre_3	0.9023	1.46%↑	0.9070	0.69%↑
ERNIE+Pre_4	0.9028	1.51%↑	0.9099	0.98%↑
ERNIE+Pre_5	0.9025	1.48%↑	0.8921	0.80%↓
ERNIE+Pre_6	0.8618	2.59%↓	0.8978	0.23%↓
ERNIE+Pre_7	0.8608	2.69%↓	0.8989	0.12%↓
RoBERTa	0.8790	-	0.8900	-
RoBERTa+Pre_1	0.8778	0.12%↓	0.8840	0.60%↓
RoBERTa+Pre_2	0.8756	0.34%↓	0.8989	0.89%↑
RoBERTa+Pre_3	0.8876	0.86%↑	0.9076	**1.76%** ↑
RoBERTa+Pre_4	0.8809	0.19%↑	0.8846	0.54%↓
RoBERTa+Pre_5	0.8776	0.14%↓	0.8901	0.01%↑
RoBERTa+Pre_6	0.8719	0.71%↓	0.8876	0.24%↓
RoBERTa+Pre_7	0.8768	0.22%↓	0.8699	2.01%↓
ChineseBERT	0.8999	-	0.9040	-
ChineseBERT+Pre_1	0.9100	0.01%↑	0.9122	0.82%↑
**ChineseBERT+Pre_2**	0.9021	0.22%↑	**0.9201**	1.61%↑
ChineseBERT+Pre_3	0.8994	0.05%↓	0.9101	0.61%↑
ChineseBERT+Pre_4	0.8804	1.95%↓	0.9043	0.03%↑
ChineseBERT+Pre_5	0.9010	0.11%↑	0.9000	0.40%↓
**ChineseBERT+Pre_6**	**0.9199**	**2.00%** ↑	0.9130	0.90%↑
ChineseBERT+Pre_7	0.8998	0.01%↓	0.9107	0.67%↑

For THUCNews the best-performing preprocessing method demonstrates that the correct Chinese word segmentation can help the model to better understand the text, enabling it to extract richer lexical-semantic information, and the division of sentences into words can help the model to differentiate the meanings of different words, and thereby improve its classification accuracy. For instance, the original text sample may be a piece of consecutive Chinese text, such as “Last night’s soccer game was very exciting and both teams performed well. (昨晚的足球比赛非常精彩, 双方球队都表现出色。)”. After word segmentation, we can divide it into a series of words, such as “last night (昨晚)”, “of (的)”, “soccer (足球)”, “game (比赛)”, “very (非常)”, “wonderful (精彩)”, “,”, “both sides (双方)”, “teams (球队)”, “both (都)”, “performance (表现)”, “outstanding (出色)”, and “。”. Then, after segmentation, each word becomes a feature of the model input, and and removing stop words can reduce the model’s attention to some common but not actually semantic words, thus reducing the influence of noise, so that some important keywords occupy a higher weight in the textual representation, which helps the model to better capture the important features of the text. That is, the stop words such as “the (的)”, “is (是)”, “both (都)”, and etc., in the categorization of sports news may not have much distinguishing power. By removing these stop words, we can get a more refined sequence of features such as “last night (昨晚)”, “soccer (足球)”, “game (比赛)”, “very (非常)”, “wonderful (精彩)”, “,”, “both sides (双方)”, “teams (球队)”, “performance (表现)”, “outstanding (出色)”, “。”.

Furthermore, removing special characters can make the sentence cleaner and clearer, which helps the model to understand the syntactic structure and logic of the sentence more accurately and helps it to avoid confusion. For example, by removing the special characters and punctuation, we can get further cleaned up feature sequences, such as “last night (昨晚)”, “soccer (足球)”, “game (比赛)”, “very (非常)”, “wonderful (精彩)”, “both sides (双方)”, “team (球队)”, “performance (表现)”, “outstanding (出色)”. These words focus on sports-related content, removing punctuation that may introduce interference. Therefore, reasonable Chinese word segmentation, stop word removal, and special character removal can each improve the performance of the general domain text categorization tasks. These preprocessing methods help a model to understand the text better, capture key information, and reduce noise interference, thus improving classification results.

For the best preprocessing method and model combination for tax question data, stop word removal and special character removal helped to remove noise, improve text clarity, highlight key information, and avoid ambiguity with Chinese-BERT in a jargon-heavy setting. The preprocessing methods help the model to understand the meaning of the tax text, which improves the accuracy and interpretability of the text categorization. For example, the original text of “According to tax regulations, taxpayers are required to complete tax declaration and payment by the end of each month. (根据税务规定, 纳税人需在每月底前完成税款申报和缴纳。)” becomes “Tax regulations require taxpayers to complete their tax returns for payment at the end of each month. (税务规定, 纳税人需每月底完成税款申报缴纳)” after removing stop words. Removing stop words such as “according to (根据)” and “need to (需)” retains important action and time information, making the sentence more concise and highlighting key information. Likewise, in the original text, “According to the second paragraph of Article 5 of the Value-added Tax Law, the sales of goods include sales revenue, taxes, surcharges, etc. (根据《增值税法》第五条第二款规定, 货物的销售额包括销售收入、税金、附加费等)” becomes “According to article 5, paragraph 2, of the VAT Law, the sales of goods include sales revenue, taxes, surcharges, etc. (根据增值税法第五条第二款规定, 货物的销售额包括销售收入、税金、附加费等)” after removing special characters. The removal of the title and pointed brackets makes the citation of the statute clearer and less intrusive with special characters, which helps the model to understand the provisions more accurately.

## Discussion

### Evaluation analysis

**Fig 2:**
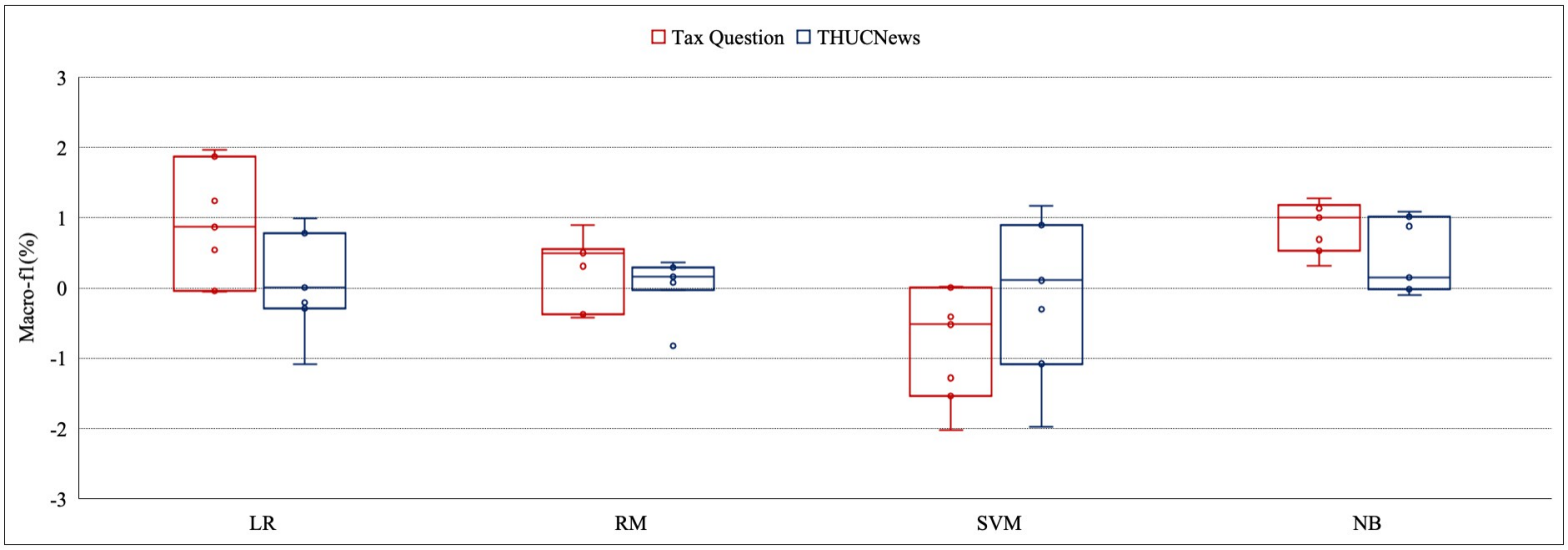
The evaluation results based on four simple machine learning models for two datasets.

**Fig 3:**
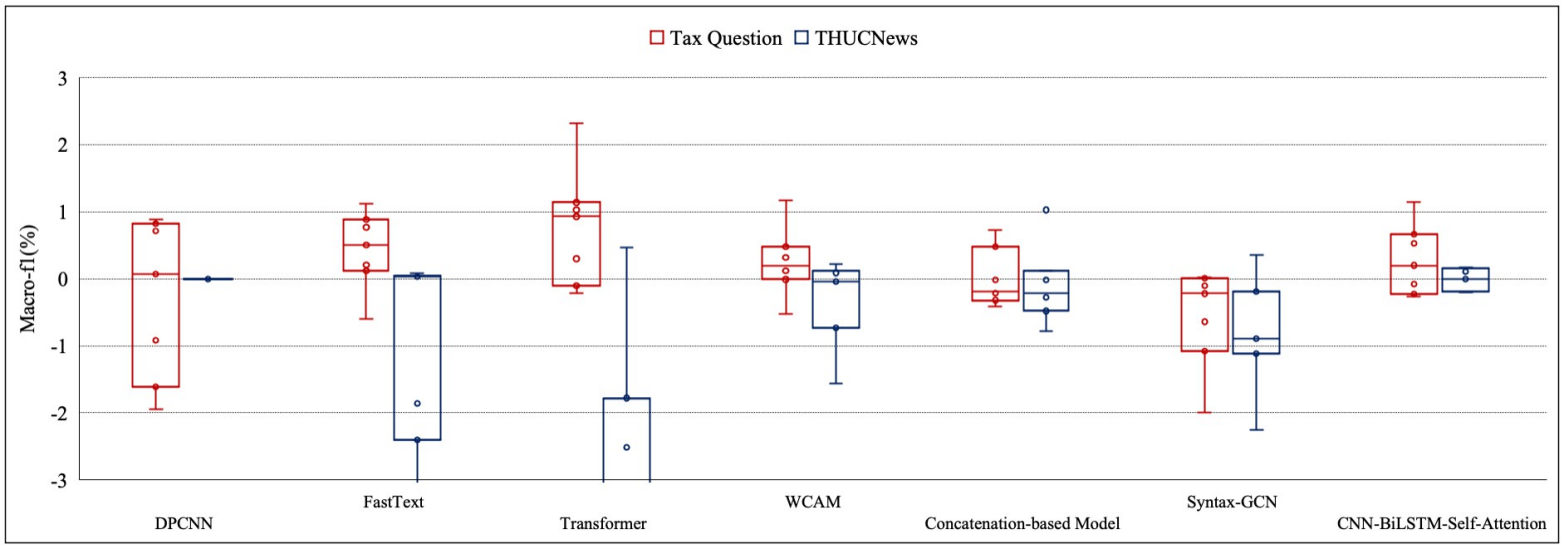
The evaluation results based on seven deep learning models for two datasets.

**Fig 4:**
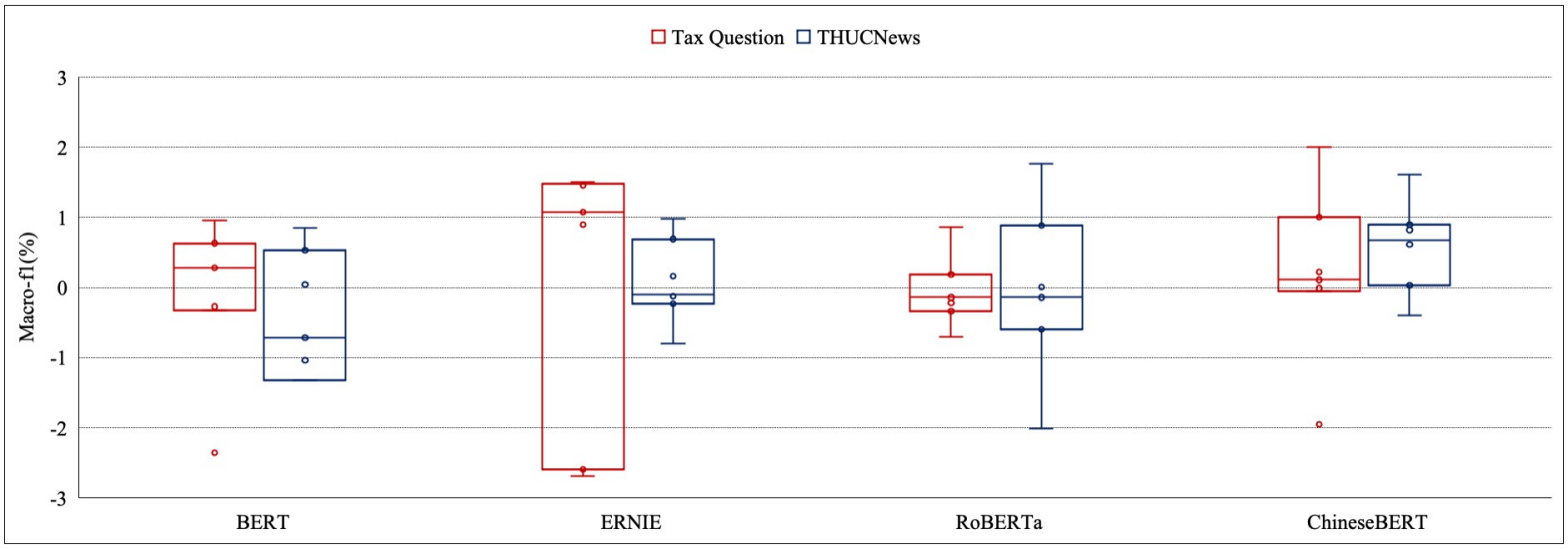
The evaluation results based on four pretraining language models for two datasets.

In this section, the macro F1 results attained by all 7 combinations of the preprocessing methods are measured to assess the preprocessing influence for Tax Question and THUCNews. The highest macro F1 among all types of classifiers and the commensurate preprocessing combinations are displayed in the following figures. According to all the models, the difference between the maximum and minimum macro F1 for all combinations of the preprocessing ranges from 0.01% to 3.28%. Moreover, the difference of maco-f1 lies between 0.01% and 1.17% for THUCNews, and between 0.01% and 1.97% for tax question based on the machine learning models in Fig 2. As for deep learning model, the macro-f1s improve from 0.01% to 3.28% for THUCNews and from 0.04% to 1.03% for tax question in Fig 3. The same type of the macro-f1s of pre-trained language model are increased by at least 0.01% and up to 2.00% for THUCNews and also at least 0.01% and up to 1.76% for tax question in Fig 4. The magnitude of differences in macro F1s proves that the congruous preprocessing combinations decide the classifiers that may meliorate the classification effect.

### Case study

**Fig 5:**
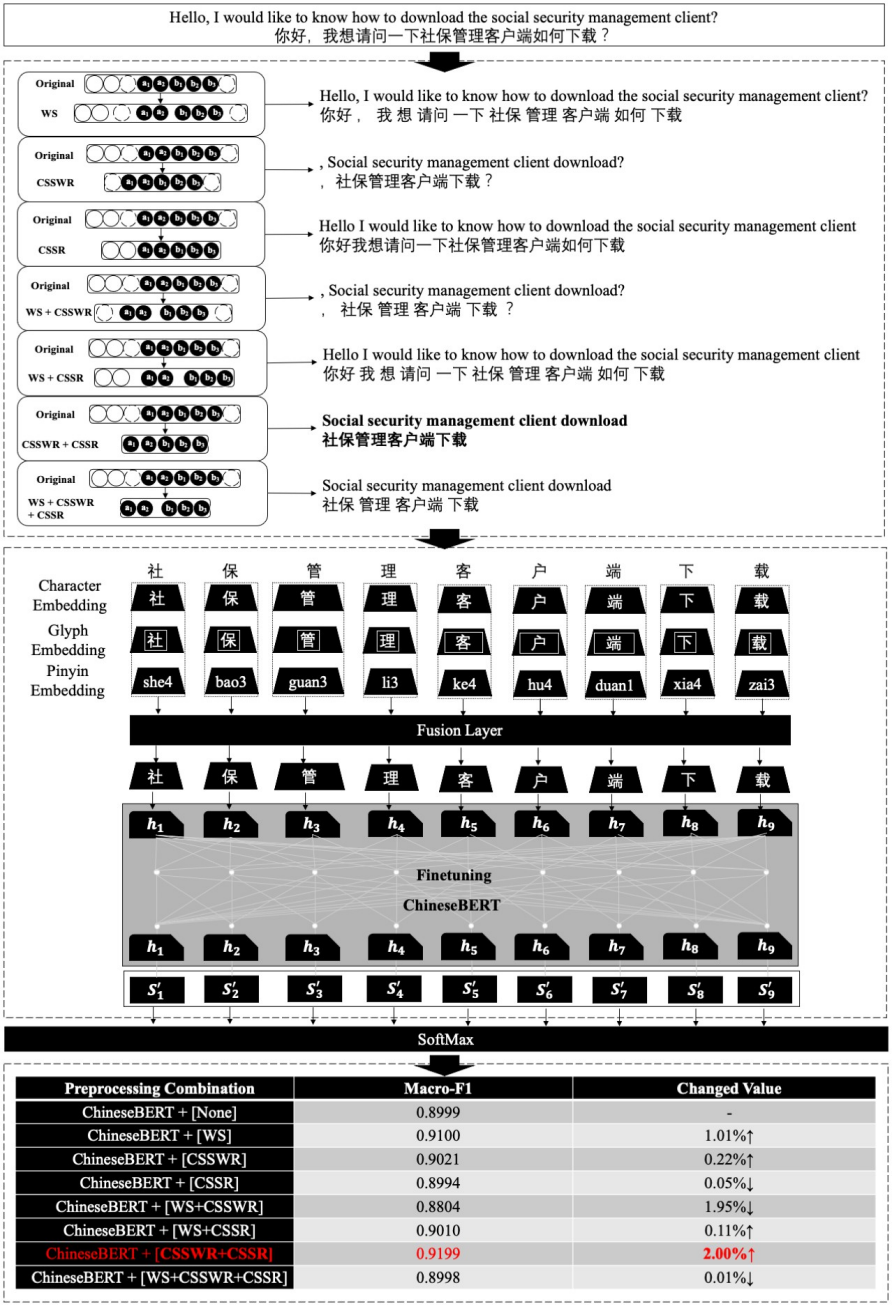
A case study of the proposed workflow in the field of taxation.

We conducted a case study for the preprocessing method for short text classification in the field of taxation, and Fig 5 shows the entire workflow. Here we see that first, the original text is processed by a combination of seven preprocessing methods, and then feature extraction is carried out in three ways, namely, TF-IDF, word2vec, and feature characterization and extraction based on a pre-trained language model. After that, feature extraction is carried out in three ways as well: TF-IDF, word2vec, and pre-trained language model-based feature characterization and extraction. Finally, the classification results are predicted by a simple machine learning model, a deep learning model, and a softmax function evaluated by f1 value, precision, and recall. Through our experimental comparison, we know that for the tax question dataset, ChineseBert produced the highest classification effect after combining with CSSWR and CSSR. This is because in the tax question dataset, its data characteristics are mainly focused on the high error rate of manual consultation, on manually generated misspellings and tone of voice auxiliaries that cannot have a positive impact on the classification results. Text content that cannot have a positive impact on the classification results can be eliminated by combining the two preprocessing methods of CSSWR and CSSR to obtain the optimal classification combination model based on ChineseBert in order to solve the semantic characterization problem of questions in the tax domain in a targeted manner.

## Conclusion

In this paper, we experimentally compared fifteen commonly used classifiers on two Chinese datasets, THUCNews and tax question datasets, which employed three widely used Chinese preprocessing methods: WS, CSSWR, and CSSR.

From the experimental results and discussion, we come to the following conclusions: we conducted a battery of various additional experiments, and found that most of the classifiers improved in performance after proper preprocessing was applied. Our general conclusion is that the systematic use of preprocessing methods can have a positive impact on the classification of Chinese short text, using classification evaluation such as macro-F1, combination of preprocessing methods such as word segmentation, Chinese-specific stop word and symbol removal, and classifier selection such as machine and deep learning models. We find that the best combination of preprocessing and classification models for categorizing text for THUCNews and Tax domain problems are FastText with WS, CSSWR, and CSSR, and ChineseBERT with CSSWR and CSSR, respectively. The macro-f1s of these methods were 92.13% and 91.99% on our tested data, which represent improvements of 0.3% and 2%, respectively over FastText and ChineseBERT themselves.

Our work provides a detailed analysis of the influence of the preprocessing methods on Chinese classifiers and fills the in-depth exploration gap of the Chinese classifier influencing factors and raises attention to the preprocessing methods used during Chinese classification. However, there are still various limitations in this work, such as the lack of preprocessing method comprehensiveness. Subsequent work will build on this foundation and provide a detailed delineation of Chinese preprocessing methods and classifiers, in order to make the research points more convincing.
