# Expression of Concern: Research on performance variations of classifiers with the influence of pre-processing methods for Chinese short text classification

**DOI:** 10.1371/journal.pone.0343161

**Published:** 2026-02-18

**Authors:** The PLOS One Editors

After this article [1] was published, concerns were raised that the article uses terminology that differs from standards in the field (e.g. “informational index” instead of “dataset”).

In addition, the *PLOS One* Editors have concerns about the article’s compliance with the PLOS Authorship policy.

In light of the cumulative issues, the *PLOS One* Editors issue this Expression of Concern. Readers are advised to interpret the article [1] with caution.
